# Assessing formal service providers’ readiness to address IPV among women with disabilities in Sweden: A mixed-methods analysis

**DOI:** 10.1371/journal.pone.0359866

**Published:** 2026-10-06

**Authors:** Cartrine Anyango, Isabel Goicolea, Fredinah Namatovu

**Affiliations:** 1 Department of Epidemiology and Global Health, Umeå University, Sweden; 2 Centre for Demographic and Ageing Research (CEDAR), Umeå University, Sweden; Independent Global Health Consultant, INDIA

## Abstract

Women with disabilities face a heightened risk of intimate partner violence (IPV), yet formal support systems often remain ill-equipped to address their specific needs. This study assesses indicators of readiness among formal service providers in Sweden, including healthcare, social services, shelters, the police, and the Centre against Violence, to support women with disabilities who experience IPV. Employing a sequential exploratory mixed-methods design, the study combined qualitative interviews (N = 18) with a national survey (N = 1,151). Data analysis was guided by an adapted version of Colombini et al.’s readiness framework, focusing on structural, organizational, and provider-level dimensions. The findings indicate that, at the structural level, reported facilitators of readiness include functional collaborative spaces, coordination, and knowledge-sharing mechanisms. However, fragmented inter-institutional collaboration and limited resources emerged as key barriers to the structural conditions required to optimally support women with disabilities subjected to IPV. At the organizational level, readiness was hindered by the absence of disability-inclusive IPV guidelines and persistent accessibility barriers, such as physical, information, and communication inaccessibility. At the provider level, many service providers expressed a need for more training on disability-specific issues, often relying instead upon personal experience or peer support when handling IPV cases. Nonetheless, supportive workplace environments and collegial networks were identified as key enablers of emotional resilience and professional learning. The findings of this study underscore the need for inclusive policies, comprehensive targeted training, and inter-sectoral collaboration to enhance the readiness of IPV support systems for women with disabilities. A truly prepared IPV support system would integrate disability-specific routines, ensure accessibility across all service dimensions, and nurture a supportive environment for IPV service providers. Addressing both systemic and interpersonal aspects is essential to improving the quality and continuity of IPV support for women with disabilities in Sweden.

## Introduction

Intimate partner violence (IPV) is a pervasive global public health issue, affecting approximately one in three women globally [1]. The consequences of IPV are profound, encompassing a wide range of physical, psychological, and social harms [2,3]. While IPV affects women across all demographics, research consistently shows that women with disabilities face a significantly heightened risk compared to women without disabilities [4–7]. This increased vulnerability is attributed to intersecting factors such as dependence upon caregivers, societal discrimination, and systemic barriers that hinder access to support services [8].

Women who experience IPV often turn to formal support systems, including healthcare, the police, social services, and women’s shelters. Evidence suggests that women experiencing IPV utilize healthcare services more frequently than those not subjected to abuse [9]. In Sweden, for instance, women who engage with social services are often more exposed to violence than the general population. However, many social workers report feeling ill-equipped to address IPV effectively [10]. The readiness of these institutions, and the professionals within them, to respond appropriately is therefore important, particularly when supporting women with disabilities.

The concept of “readiness” is central to understanding how well formal support systems can respond to IPV. Readiness refers to the willingness and ability of individuals or organizations to implement specific interventions or innovations [11,12]. It operates across multiple levels, including individual providers, teams, departments, and entire organizations [12]. Readiness encompasses several dimensions: the knowledge, attitudes, motivation, and emotional preparedness of service providers [13,14]; the structural capacity of services, including infrastructure and resource availability [15]; and the broader organizational culture and governance frameworks that support implementation [12].

Research on readiness to respond to IPV has largely focused on healthcare professionals [14,16–19]. For instance, healthcare professionals’ readiness to address IPV in their daily practice hinges on their attitudes and beliefs about IPV, as well as their perceived preparedness and actual knowledge and skills about how to handle IPV victim-survivors [17]. Studies on readiness exist in other sectors as well. In Australia, for example, studies have explored the readiness of graduate and hospital social workers to recognize and respond to domestic and family violence [20,21]. While not explicitly framed as “readiness”, related research in Sweden has explored the concept of quality in IPV shelters [22], which is a critical component of organizational readiness. High-quality services are essential for ensuring that IPV victim- survivors receive effective support.

Effective IPV support requires readiness at structural, organizational, and provider levels [19]. However, various barriers – including inadequate training, societal stigma, and limited time and resources – can hinder the ability of professionals to effectively support IPV victim-survivors [23,24]. Effective IPV support requires the integration of IPV response into the broader range of services that women commonly access, such as physiotherapy sessions or routine health screenings. It also demands that service providers possess key interpersonal skills, including empathic listening, validation, and the ability to offer appropriate referrals [25]. Systemic changes are also necessary, including a shift from a narrow medical model to a more holistic, care-oriented approach [13], and structural reforms that challenge the normalization of IPV [26]. Furthermore, delivering effective IPV support depends upon adherence to best practices and robust communication skills. Providers must be able to recognize or suspect signs of violence and engage with women in a sensitive, non-judgemental manner, acknowledging their experiences, respecting their choices, and connecting them with the necessary support services [27].

For women with disabilities, additional challenges further compromise service providers’ readiness. These include communication barriers, lack of disability-specific training, and pervasive prejudices against people with disabilities [28]. Enhancing readiness in this context requires tailored communication tools, specialized training, and the cultivation of trust between providers and survivors [28].

At the organizational level, readiness is shaped by systemic factors such as funding limitations, inadequate intersectoral coordination, and the physical inaccessibility of services [29,30] for women with disabilities. A recent systematic review highlights that structural reforms are essential to improve organizational readiness, including the allocation of resources, implementation of inclusive policies and guidelines, and ensuring accessibility for women with disabilities [30].

Despite their increased vulnerability, women with disabilities often encounter significant barriers when seeking IPV support. Service providers often struggle to recognize signs of abuse among this group [31], while women with disabilities report facing stigma, discrimination, social exclusion, and physical obstacles to accessing IPV support services [32]. Research indicates that support for people with disabilities is also hindered by a lack of awareness, training, and resources [28], suggesting inadequate readiness among both the support system and individual providers.

Despite these insights, there is limited knowledge about the readiness of formal support systems to provide support to women with disabilities, particularly across different formal support sectors. This study aims to fill this gap in knowledge by assessing indicators of service providers’ readiness to offer support to women with disabilities subjected to IPV in Sweden, across structural, organizational, and provider dimensions.

### Conceptual Framework

This study draws upon the conceptual framework developed by Colombini et al. [33] for improving health-system readiness to address violence against women and girls [33]. This framework offers a comprehensive and structured approach to assessing the capacity of systems and service providers to deliver effective support to IPV victim-survivors [19].

The framework identifies three interrelated domains of readiness: structural readiness (at the macro level), organizational readiness (at the meso level), and provider readiness (also at the meso level) [33]. At the macro level, structural readiness refers to the broader systemic and policy environment that enables or constrains IPV response. This includes the presence of a robust policy framework, governance structures, and accountability mechanisms that support the integration of IPV services across sectors. Structural readiness is critical for ensuring that IPV interventions are not only prioritized but also sustainably implemented within formal support systems [33].

The meso-level of the framework focuses on organizational and provider readiness. Organizational readiness encompasses the internal culture, structures, resources, and operational processes of institutions. It also includes the extent of coordination and communication across different levels within an organization. Provider readiness, on the other hand, refers to the knowledge, skills, attitudes, and emotional preparedness of individual service providers to respond effectively to IPV cases. Together, these dimensions (provider and organizational readiness) determine whether institutions are functionally equipped to deliver accessible, coordinated, and survivor-centred IPV support [33].

In this study, structural readiness includes factors such as the allocation of resources for accessible, inclusive, and equitable IPV services, and the degree of intersectoral collaboration among key institutions (healthcare, social services, the police, and women’s shelters). Effective coordination across these sectors is essential for delivering a comprehensive and integrated response to IPV.

At the organizational level, readiness is assessed in terms of how well IPV services are adapted to meet the diverse needs of women with disabilities, including those with mobility, sensory, or cognitive impairments. This involves ensuring that services are physically, cognitively, and communicatively accessible. Examples include the availability of ramps, sign-language interpreters, easy-to-read materials, and assistive technologies that support communication and mobility. Organizational readiness also includes the presence of clear guidelines, protocols, and routines that institutionalize inclusive and responsive IPV support services.

Provider readiness focuses on equipping professionals with the necessary knowledge and competencies to support women with disabilities subjected to IPV. This includes training on both IPV dynamics and disability inclusion, as well as the development of internal support mechanisms such as mentoring, peer networks, and reflective supervision. These structures help to sustain provider motivation and emotional resilience, which are crucial for delivering empathetic and effective IPV support.

While Colombini et al.’s framework was originally developed to strengthen health‑system readiness in lower‑and middle‑income settings, its core domains: structural, organizational, and provider readiness; are conceptually transferable across service systems. For this study, the framework was adapted to a high‑income, multi‑sectoral context by expanding structural readiness to include intersectoral governance and coordination across formal IPV support institutions. Organizational readiness reflects accessibility and disability‑inclusion standards that characterize high‑income welfare systems, and provider readiness was broadened to encompass the competencies of diverse frontline professionals, not only health‑care providers. These adaptations retain the original conceptual structure while ensuring relevance to the Swedish IPV service system. Table 1 (below) outlines the components of the adapted readiness framework used in this study, encompassing structural, organizational, and provider readiness at the macro and meso levels. While this study focuses on these two levels, it is important to note that the original framework by Colombini et al. [33] also includes client and community readiness, which are beyond the scope of this research.

**Table 1:** Components of the adapted readiness framework for IPV support systems.

Readiness category	Key components
Structural readiness (Macro level)	Overarching policies Governance Resource allocation
Organizational readiness (Meso level)	Protocols and guidelines Availability of resources, time, infrastructure, equipment Supportive and effective management Supportive organizational culture
Provider readiness (Meso level)	Staff capabilities: knowledge, confidence Support system in place, mentorships, supervision, team networks

## Materials and Methods

This study employed a sequential exploratory mixed-methods design [34–36], integrating qualitative and quantitative approaches in order to assess indicators of readiness across formal support systems for supporting women with disabilities subjected to IPV.

The study was conducted in two phases. The qualitative phase preceded the quantitative phase, allowing insights from the former to inform the development of the survey instrument.

This design enabled the strengths of both methods to be leveraged: the qualitative data provided an in-depth understanding of service providers’ experiences and perspectives, while the quantitative data helped to identify broader patterns across a larger sample. Together, they contributed to a holistic understanding of readiness across formal support institutions.

### Qualitative phase

#### Participant recruitment and data collection.

In this initial phase, we purposively recruited 18 professionals from diverse formal support institutions across Northern Sweden. These included healthcare (N = 8), social work (N = 3), the police (N = 4), women’s shelters (N = 2), and the Centre against Violence (N = 1). Recruitment of participants began in early April 2020, with the first interview conducted on 15th April 2020. The recruitment and data collection process continued until the final interview was conducted on June 18th, 2020. We officially announced the completion of the data collection process on February 18th, 2021. Participants were selected on the basis of their direct experience in working with women with disabilities subjected to IPV, ensuring relevance and depth of perspective. Interviews were conducted by one member of the research team, in Swedish, guided by a semi-structured interview guide. Participants’ professional experience ranged from two to 20 years, reflecting a wide range of professional expertise.

### Qualitative data analysis

To begin with, after the interviews, we performed a preliminary data analysis. The interviews were transcribed verbatim, translated into English, and analysed using reflexive thematic analysis, a flexible approach that emphasizes the researcher’s active interpretative role in theme development [37,38]. The analysis was not comprehensive at this preliminary stage, but the themes developed – including aspects of collaboration, training, accessibility, guidelines and routines – were then used to inform the design of the subsequent cross-sectional survey instrument.

The second, more comprehensive, data analysis was performed after the descriptive analysis of the survey data. The first author revisited the qualitative dataset, which was imported into MAXQDA, a qualitative data analysis software package, to further facilitate the data analysis process. This time, the analysis was deductive, guided by both the conceptual framework on readiness and findings from the quantitative descriptive analysis. The quantitative findings on various aspects of readiness – including training, routines and guidelines, service accessibility (etc.) – guided the coding and thematization process, applying the readiness framework as a lens to assess structural, organizational, and provider-level dimensions. The analysis process was iterative, involving continuous comparison between qualitative insights and quantitative patterns. By the end of this process, the qualitative data had provided rich contextual explanations that complemented and deepened the interpretation of the survey results. Table 2 provides an overview of how the preliminary qualitative themes informed the construction of survey items and how both qualitative (QUAL) and quantitative (QUAN) datasets were subsequently integrated during the interpretation of findings.

**Table 2:** Overview of phase1 informing phase 2 and integration.

Preliminary qualitative themes (Phase 1)	Corresponding survey items (Phase 2)	Integration during analysis (QUAL and QUAN findings were complementary)
**Structural readiness**		
Collaboration and coordination across institutions	Intersectoral collaboration	Quantitative patterns used to identify collaboration across institutions; qualitative data used to explain variations in how collaboration is practiced and specificity on the intra and inter institutional collaborations
Workload, resources, and structural constraints	Not captured in survey	Qualitative data provided detailed examples of funding instability, workload pressures, and data system limitations
**Organizational readiness**		
Availability of guidelines and routines	Items on availability of IPV and disability-specific guidelines and routines	Survey frequencies linked with interview insights about gaps, inconsistencies, and practical challenges in applying guidelines
Accessibility of services (physical, information, communication)	Items on physical accessibility, accessible information, availability of communication tools	Qualitative narratives elaborated on practical accessibility barriers, enabling interpretation of low survey percentages
**Provider readiness**		
Organizational culture and support (supervision, peer networks)	Items on availability of someone at work to consult	Interviews contextualized survey findings, illustrating how workplace culture influenced emotional resilience and readiness through collegial support, supervision, and managerial support
Training needs and capacity gaps	Items on IPV training, disability-specific training, and need for more training	Qualitative accounts used to explain training deficits identified quantitatively and show variations across sectors

In this study, integration occurred at three points. At the design stage, preliminary qualitative themes determined the content of the survey items (Table 2). At the analysis stage, the descriptive survey findings structured a second, deductive pass through the qualitative dataset, with the readiness framework as the organizing lens. At the interpretation stage, the two strands (QUAL and QUAN) were brought together within each readiness domain: for each indicator, the survey distribution was placed alongside the corresponding qualitative accounts, and the two were examined for convergence, complementarity, expansion, or contradiction. Where the strands addressed the same phenomenon at different levels of specificity, the qualitative material was given interpretive priority for explaining how and why a condition arose, and the quantitative material for establishing how widely it was reported.

### Quantitative phase: Data collection and analysis

#### Survey development.

Insights from the qualitative phase directly informed the development of the survey instrument; the correspondence between the preliminary qualitative themes and the survey questions is set out in Table 2 above, and the operationalization of readiness indicators is described below. The survey targeted professionals from healthcare, social services, and the police. Professionals from the women’s shelters and the Centre against Violence were not included because Statistics Sweden (SCB) did not hold their contact information.

### Participants and sampling

SCB identified potential participants using data from the 2020 occupational register. The sampling frame was stratified into 13 occupational strata (see our previously published work for more details on these strata) [39], from which a random sample of 3,500 individuals was drawn, ensuring an equal selection probability within each stratum. After excluding eight individuals due to emigration or death, the final sample consisted of 3,492 professionals, all of whom were invited to participate in the survey.

### Data Collection

SCB conducted the survey on behalf of Umeå University. Prior to distribution, the questionnaire was piloted, and feedback was used to refine its content and structure. The final survey was administered between September and November 2022. Participants received the questionnaire by mail, followed by three reminder mails/posts to encourage participation. Out of the 3,492 service providers contacted, a total of 1,151 responded, resulting in a response rate of 32.9%, which is consistent with other SCB-administered surveys. Among the respondents, 81.3% were women, and 61% worked in the healthcare sector. Table 3 presents the demographic and professional characteristics of the survey participants.

**Table 3:** Participants’ demographic characteristics (N = 1151).

Variable	N (%)
	
**Age-group**	
24–44	419 (36.4%)
45–64	557 (48.4%)
65+	175 (15.2%)
	
**Gender**	
Male	215 (18.7%)
Female	936 (81.3%)
	
**Country of Birth**	
Sweden	1036 (90.0%)
Outside Sweden	115 (10.0%)
	
**Occupation**	
Police	64 (5.6%)
Social services	182 (15.8%)
Healthcare	706 (61.3%)
Others	198 (17.2%)
Missing	1 (0.1%)
	
**Years Worked (Years of Experience)**	
0–5 years	171 (14.9%)
6–9 years	169 (14.7%)
More than 10 years	605 (52.6%)
Missing	206 (17.9%)
	
**Type of Employment**	
Permanent Employee	844 (73.3%)
Temporary employee	18 (1.6%)
Project/Hourly employee	40 (3.5%)
Others	46 (4.0%)
Missing	203 (17.6%)

### Data Analysis

We performed a descriptive analysis of the survey responses from these 1,151 service providers, using Stata version 14.2. We assessed key readiness indicators, including availability of guidelines and routines, services accessibility, training, and employer support, alongside demographic characteristics. After this analysis, we revisited the qualitative dataset to further explore and contextualize the descriptive quantitative findings. This iterative approach enabled a nuanced interpretation of the data, enriching statistical trends with qualitative depth and insight.

### Operationalization of readiness indicators

The survey questions were developed from the preliminary qualitative analysis (Table 2) and were subsequently read through the readiness framework during interpretation. Structural readiness was represented by intersectoral collaboration; organizational readiness by guidelines and routines for supporting people with disabilities subjected to IPV, physical accessibility of the workplace, accessible information, and communication tools such as hearing loops and sign-language interpretation; and provider readiness by IPV training, disability-specific training, the expressed need for further training, and the availability of a colleague or supervisor to consult.

To our knowledge, no validated instrument currently exists for assessing disability-inclusive IPV readiness across multiple formal support sectors. Because each dimension was captured through discrete, single questions rather than multi-item scales, internal-consistency statistics such as Cronbach’s alpha were not applicable, and no composite readiness score was constructed. Two consequences follow for interpretation. The indicators describe the presence or absence of particular readiness conditions rather than the magnitude of readiness, and because they were neither weighted nor combined, we do not claim that they exhaust the readiness construct. Throughout this paper, therefore, we refer to indicators or dimensions of readiness; readiness itself is interpreted through the integration of these indicators with the qualitative material and the conceptual framework.

### Ethical considerations

The study obtained ethical approval from the Swedish Ethical Review Authority [J. Reg no. 2019–05249].

### Qualitative phase

Participation in the qualitative phase was entirely voluntary. Informed consent was obtained both verbally and in writing prior to data collection. Potential participants were initially contacted and provided with an information letter outlining the study’s purpose. Before each interview, the interviewer (a member of our research team) reiterated the study’s objectives, addressed any questions, and emphasized that participation was optional and that the participant could withdraw at any time without consequence. The verbal consent was then sought at this point and recorded. To ensure confidentiality, all personally identifying information was anonymized during the transcription and analysis processes.

### Quantitative phase

For the survey phase, participants received an introductory letter explaining the study’s objectives and the collaboration between Umeå University and SCB. The letter highlighted the voluntary nature of participation and clarified that certain demographic data would be retrieved from SCB’s registers in anonymized form. It also outlined the strict confidentiality protocols and data protection agreements governing the handling and sharing of data between SCB and Umeå University. Only those willing to participate logged in and answered the digital questionnaire.

## Results

The findings integrate quantitative survey data with qualitative interview insights and are organized according to Colombini et al.’s [33] framework, which includes three interrelated domains of readiness: structural readiness, organizational readiness, and provider readiness.

### Structural readiness

According to Colombini et al. [33], structural readiness refers to the broader systemic and policy-level capabilities that shape the implementation of IPV support services within any given institution. These include governance structures, intersectoral collaboration, and resource allocation. In our study, from the analysis, we identified two structural dimensions shaping readiness: 1) the functionality of collaborative spaces, and [2] resource and operational constraints.

### (Non)functional collaborative spaces

The quantitative findings showed that slightly more than half (57%) of the surveyed service providers highlighted collaboration as a key workplace aspect in supporting people with disabilities subjected to IPV. However, the qualitative data revealed variations in how collaboration was practised across institutions. Some professionals described active engagement in inter- and intra-institutional networks, such as regular multidisciplinary meetings and team reflections upon violence against people with disabilities. These spaces were seen as valuable for continuous learning and coordination:

*When we meet in that network, it’s violence most of the time… We’ve been working on material in our workplace, it’s short films that we watch together as the whole team, so we reflect and have slightly different questions about what we see. Then it’s based on people with different disabilities and how they’re in a position of dependence […]. It’s been a great feature to have in the whole workplace because good conversations will come out of them. Yes, I think we have a pretty good foundation to stand on. These recurring invitations to networks and training that relate to this area [violence against people with disabilities], we get to take part in them. It’s valuable because you’re constantly replenishing with new knowledge and with new information. (SP3, Healthcare)*

Other service providers emphasized the inter-institutional collaborations that they have established to respond to women with disabilities subjected to IPV. One social worker (SP4) pointed out that these collaborations are sometimes based on the victim’s disability, which can result in more collaboration with other institutions than with others.

*We collaborate with the police, the healthcare system, and then it [collaboration] also depends on what disability one has, psychiatry is pretty common. It’s almost the department that we collaborate with the most within healthcare. (SP 4, social work)*

Others described their inter-institutional collaborations with certain support institutions, emphasizing that these collaborations are sometimes based on the needs of the woman seeking support and sometimes on their disability status. However, these collaborations were not with all the institutions. One police officer (SP6) provided an explanation of the collaborative efforts, cooperation, and referrals that they undertake.

*Of course, we cooperate with social services when there’s a need for support, or you refer and cooperate to a certain extent with women’s shelters, or you get information from the healthcare,* Barn- och ungdomspsykiatri *(BUP) [Child and Adolescent Psychiatry], or some psychologist or someone. But I wouldn’t say that we’re cooperating with civil society organizations. (SP6, Police)*

Others reported fragmented collaborations lacking formal feedback loops, which hindered continuity of support and follow-up on referrals. One service provider from the social work sector (SP2) perceived this as hindering the continuity of IPV support coordination and posing difficulties in following up or determining the outcome after referring a victim to other support institutions:

*Feedback is bad. I’ve sent two off to another place, for example. And then you wonder, how did that turn out then? [….]. But we don’t get any feedback unless the person herself continues to keep in touch with me. (SP2, Social work)*

### Resources and operational constraints

Although not directly addressed in the survey, the interviews highlighted structural challenges related to unstable funding, workload pressures, and inefficient data systems. These constraints were not limited to disability-specific IPV support but reflected broader structural issues.

Service providers, particularly those from the women’s shelters, expressed concerns about the lack of sustainable funding. Many relied on short-term grants, which created uncertainty and limited long-term planning:

*Yes, we want safer funding, and so do all the emergency services in our country all the time. We want more stable and longer-term cooperation with our municipality, for example, which would mean safer financing for part of our work anyway, that would be nice. Then it’s about applying for grants for each year on several occasions, e.g., government grants or public health grants, the National Board of Health and Welfare sometimes announces different types of funds for different times of the year, so our municipality is of course only part of it, but an important part. (SP1,Women’s Shelter)*

Another aspect highlighted by the women’s shelters was the issue of workload. Women’s shelters in Sweden are often run voluntarily and are one of the most common places where female victim-survivors of IPV seek safety. This could explain the workload issue. Additionally, disability and IPV can potentially worsen time allocation. For instance, in terms of workload, a certain number of clients must be seen per day, which is calculated based on the “able-bodied/minded” population. However, people with certain disabilities, especially those that limit communication and understanding, require additional time. Simultaneously, IPV also requires more time, which could potentially lead to a woman with a disability subjected to IPV spending significantly more time during the process of seeking support. This situation presents a challenge for service providers in effectively managing their workload. A social worker from the women’s shelter emphasized this point:

*I lack the time to be able to take a person by the hand and walk her off to the medical centre as often as I would like. Or go to the bank and get a bank card for her. Or help [her more], because these particular women are in a dilemma that they still have things in their old home that still belong to the former partner [perpetrator]. (SP2, Social work)*

IPV case-tracking was perceived as a challenge due to what were described as inefficient data systems. One social worker (SP4) provided a scenario in which their data management systems poorly capture IPV-related cases, resulting in underreporting and difficulty tracking cases. The service provider explained it as follows:

*Because of all the applications that come in, someone doesn’t say on her first call that it’s about violence. But she might be applying for housing, or yes support, substance abuse support or something else. Then the time after, with the administrator, it comes to light that there’s been violence. We have numbers, and we keep up statistics, but it’s a big dark number...Yes, we have a bad computer system. We’re going to key in case time, I think it says at the first contact, and then if she’s new, she ends up in the reception unit and then she applies for accommodation, then they link the case to the accommodation. Then she ends up as an accommodation case in the statistics, do you understand? It can go wrong right from the start. And we’ve tried to run it manually to get better numbers, but that’s also been difficult due to the workload. (SP4, Social work)*

These findings highlight how structural-level factors – such as fragmented collaboration, unstable funding, and inadequate data systems – can undermine the readiness of formal support systems to respond effectively to women with disabilities experiencing IPV. These structural limitations also shape the organizational environments within which service providers operate, and this is the focus of the next section.

### Organizational readiness

Organizational readiness encompasses the culture, structures, and processes within institutions that support victim-survivors of IPV. In this study, this domain focuses on the availability of disability-specific guidelines, accessibility, and operational adaptations.

### Disability-specific guidelines and routines

Service providers recognized the importance of having disability-specific routines and guidelines to support people with disabilities subjected to IPV. The quantitative data indicated that 48% of service providers considered the availability of such routines essential for providing optimal support.

The qualitative interviews revealed inconsistencies in the availability and specificity of these guidelines. Some service providers noted that their institutions had dedicated guidelines for people with disabilities subjected to IPV, while others described a perception of relying upon generalized IPV guidelines. One service provider from the women’s shelters (SP1) mentioned:

*There’s a chapter in our on-call book that you can read under the study circle. It deals with violence against people with disabilities because we often encounter extra-vulnerable groups. The book covers different groups and types of violence, so that’s part of it. Apart from this chapter, there’s nothing else. (SP1,Women’s shelter)*

These perceptions varied across different institutions. One service provider from the healthcare sector (SP13) explained their perception that, while general IPV protocols and training programmes are available, they are not explicitly designed to support people with disabilities subjected to IPV.

*Yes, we have them. We have a care programme for threats and violence in the county council or the region as a whole. Our staff are constantly being trained in these issues. We’re trying to further our education. (SP13, Healthcare)*

### Information accessibility and support fragmentation

When asked about the measures being taken to improve accessibility for people with disabilities subjected to IPV, only 42% of service providers reported having accessible information at their workplaces. Qualitative findings suggested inconsistencies in their implementation and effectiveness. Some service providers described efforts to improve information dissemination, such as brochures and online materials. Nevertheless, these initiatives were perceived as often failing to account for the diverse communication needs of women with disabilities victim-survivors of IPV. One service provider from the women’s shelters (SP12) shared their approach, which involves creating an easily understandable brochure about their services and applying for funding to enhance their website’s accessibility. While acknowledging the difficulties in accommodating diverse disability needs, SP12 also mentioned their commitment to continuous improvement:

*We’ve produced an easy-to-read brochure about what we do. We’ve also applied for funding to make our website accessible with an audio-read function. But it’s difficult because everyone has different needs, and we can’t cover everything… Yes, but we’re working on that over time.* *(SP12, Women’s shelter)*

One social worker (SP4) described what she believes the IPV support for people with disabilities should look like: a comprehensive, round-the-clock (reachable all day long) service that offers immediate, dedicated support to women with disabilities subjected to IPV. In contrast, the current system, as SP4 described it, is *“for those who have more capabilities”*, which SP4 further said means those victims who are *“able to look up a number, able to Google” […]”* However, if you’re in a violent relationship, you might need to *“sneak into the bathroom to be able to call”*, which many women with disabilities cannot do.

*I wish we had an anti-violence centre where we have income support, we have child welfare officers, we have adult caseworkers, and we have conversational support […] and that applied to all the victims of violence, including disability. And so, I wish we had an on-call service where we had it around the clock, the week around. So that people who can’t live by themselves, could be allowed to seek shelter as well.* (*SP4, Social work)*

People who cannot live by themselves, as mentioned by SP4 above, include people with disabilities who may not be able find enough privacy to seek support, if there is always someone else occupying a similar space. SP4’s quote above suggests that a challenge arises due to the fragmentation of support, which they perceived as a difficulty in navigating the various formal support institutions.

### Physical accessibility

In the quantitative survey, only 47% of service providers stated that their workplaces were physically accessible (e.g., having ramps, lifts, people with disabilities’ toilets). This was echoed in the qualitative findings, where some service providers noted significant barriers to physical accessibility.

For example, one social worker (SP4) described the inaccessibility of women’s shelters:

*I think, there [physical accessibility] we’re also really bad, because none of these women’s shelters we have in [town’s name withheld] are adapted based on wheelchairs, for example. Everyone has their rooms upstairs, there are no elevators. They’re accommodated in old houses that have been rebuilt. I had a woman now who was rheumatic, and I mean it’s not, well... It’s not the worst disability, she could walk. But she can’t handle being at the women’s shelter because of the stairs and such.* (SP4, Social work)

Even when accessible housing was available, it was often limited:

*Two years ago, we acquired an external apartment for women with disabilities, but it’s often full. If someone is already living there and another woman in crisis needs accommodation [housing], we don’t have any alternatives.* *(SP12, Women’s shelter)*

Additionally, the lack of accessible transport was also described as a barrier that prevented women with disabilities from reaching the available support. One service provider from healthcare (SP7), described a scenario in which physical inaccessibility outside of the institutions also influences support provision:

*If you have a disability, you can’t just run away or drive yourself. You have to call a taxi, and there’s often no transport available at night.* *(SP7, Healthcare)*

### Communication accessibility

Only 19% of service providers reported having communication tools available such as hearing loops and sign-language interpreters. The qualitative interviews also revealed that communication barriers were a challenge, particularly for deaf and those with intellectual disability.

One healthcare service provider (SP3) recounted the challenges faced by a woman with an intellectual disability who reported IPV to the police but was unable to provide the necessary detailed evidence for legal action, resulting in the case being dropped:

*She did everything right – she reported the abuse – but because she couldn’t describe the details in a way the police needed, the case didn’t move forward. That was frustrating.* *(SP3, Healthcare)*

This quote from SP3 implies that it was not the disability itself (as it seems to suggest), but rather the lack of tools that hindered communication, particularly articulation. Consequently, the inflexible system could not allow the case to progress.

Another service provider (SP12) highlighted a lack of awareness about the availability of communication tools:

*We didn’t even know we could request a sign-language interpreter. It’s been a learning process for all of us.* *(SP12, Women’s shelter)*

The absence of accessible communication tools and the barriers associated with them were perceived to hinder not only the victim-survivors’ ability to report IPV but also their access to the services they required, such as legal aid, social services, and healthcare:

*She couldn’t call the social insurance office by herself or visit the tax office to request protected personal information. Even if she had, they wouldn’t have understood sign language. She’s had numerous physical injuries from the violence she’s endured, so we’ve had to accompany her to various doctor’s appointments and try to explain what she can’t express verbally. Despite having an interpreter with us, communication remains challenging, especially when using sign language.* *(SP12, Women’s shelter).*

Here, it is seen that communication barriers remain an obstacle, limiting service providers’ ability to provide support but also preventing the women with disabilities subjected to IPV from accessing optimal support. These findings underscore the importance of addressing organizational-level accessibility barriers to improve the readiness of support systems for women with disabilities subjected to IPV. The next section focuses on provider readiness, examining the knowledge, skills, and attitudes of service providers.

### Provider readiness

Colombini et al.’s [33] framework examines knowledge, skills, training, perceived challenges, supervision, ongoing support, and the support system, including mentoring, supervision, and team networks at the provider level. Our study found perceived knowledge gaps in training for IPV and disability-related support. Participants also described a supportive workplace environment cultivated by colleagues, managers, and supervisors, as well as inter-collegial networks and consultations.

### Staff training and capacity-building

When asked whether they felt the need for more training on providing IPV support to those with disabilities, almost two thirds (64%) of service providers expressed a need for additional training. The qualitative findings also depicted this need for more training. One service provider (SP13) explained this need:

*Yes, training is always needed. You need to constantly update yourself, and it feels important.* *(SP13, Healthcare)*

A social worker (SP4) further described the need for training, especially for IPV victims with intellectual disabilities:

*I don’t think there’s much difference between being with or without a disability. Some psychiatric diagnoses can do that extra, but intellectual disability is very special. And, for me, it’s not something we have any direct training in either; for me, it’s like investigating children. Which I’ve done too, and then I think that’s about what’s hardest too. For me, it feels like it’s children who are victims of violence. (SP4, Social work)*

SP4’s quote above suggests that training should also consider various different disabilities, because the needs of each individual vary depending on their specific disability.

Disability-specific IPV training was limited, with only 18% of service providers reporting formal training on disability support for people with disabilities who are victims of IPV. Instead, most service providers relied upon their own experience when it came to disability-related IPV cases. One service provider from a women’s shelter (SP1) described this reliance upon experience:

*We don’t have formal training on disability, but we know about violence, which is our area of expertise. We understand how violence manifests and how it works, but we don’t know much about disabilities except what we learn from our experiences. I would say that’s our current knowledge. (SP1, Women’s shelter)*

These perceptions of a lack of disability training were echoed across several support institutions. A police officer (SP8) also shared similar sentiments:

*No, I wouldn’t say that [we have disability training]. We sometimes go to occasional lectures. It can also be web-based [training] that you listen to. We read by ourselves and try to collect information in each individual case if there’s something we need to know about the person. We have basic further training in terms of methodology, investigating violence in close relationships, but not specifically in terms of people with disabilities... I don’t think there’s anything in our education catalogue that specifically relates to people with disabilities. I haven’t seen that anyway… It’s more violence than disability that education contains, so to speak. (SP8, Police)*

Recognizing this gap in knowledge, some service providers reported taking independent steps to collaborate with disability organizations to enhance their understanding and their ability to provide inclusive support. One service provider from a women’s shelter (SP12) described this initiative:

*We felt that, specifically regarding disability, we need to improve and contacted functional rights [HSO or Funktionsrätten] so we could collaborate with them. Since then, we’ve involved more actors, but it’s an important matter because we want to develop, but when there aren’t any specialized resources for violence and disability, it feels important to try to get it from another source and organize it ourselves and get this training. (SP12, Women’s shelter)*

In contrast, 43% of service providers reported having participated in IPV general training. This slightly wider coverage than the disability training (18%) was also evident in the qualitative material. A service provider from the women’s shelters (SP1) described their wide experience with IPV support:

*We have long experience for 40 years. We have a lot of experience with violence. When it comes to our competence for violence, I think it’s very good and perhaps where you can get the most knowledge about violence. (SP1, Women’s shelter)*

A social worker (SP2) described formal IPV training programmes provided through municipal and government initiatives, ensuring that they had the necessary skills to identify and respond to IPV cases:

*Based on the directives that come from the government, we’ve attended training courses in [town’s name withheld]. We’ve done internal training and some stuff like that in [our] municipality at the social services because we want to get better at asking [about IPV]*. *(SP2, Social work)*

However, not all service providers had access to formal IPV training, some reported having to rely upon peer support due to the lack of recent training opportunities. One healthcare provider (SP5) described this reliance on peer support:

*It hasn’t been much [training on IPV]. Now I’ve only been here for five years. But there hasn’t been any specific training or courses or anything like that. It was last autumn when I took part in one called “Tearing down obstacles”. Then we participated in it via the web. But then we’ve also had a lecture on honour-related violence and oppression, must be about a year ago. We don’t have any tutorials. It’s been two years since we finished our tutorial. Without it, the support of my teammates at the moment is the order of the day*. *(SP5 Healthcare)*

(Non-)supportive workplace environment

When asked whether they have someone at work to consult regarding support for people with disabilities subjected to IPV, 56% of service providers stated that they did have access to such support. The qualitative findings revealed the nature and availability of such support. Many service providers perceived their peers or colleagues as valuable resources for consultation and support, especially when handling IPV cases, including the ones they referred to as *“difficult cases”*. One police officer (SP6) emphasized the importance of peer support and a positive work environment:

We *talk to each other a lot. Colleagues are probably the biggest support we have. We have a very good climate in our group where we can talk to each other. We usually do that after these interrogations in the investigations, how you experienced it. Then we’re resources for each other. We receive regular supervision in groups. It can look a little different. But if you need to talk specifically about a case, you can do it on a group basis or at individual supervision, so you can talk about what you’ve experienced, or if one case has been more difficult than another. That possibility exists. What I notice in these types of cases, anyway, is that recovery is required between interrogation and duties, because they can take quite a lot of energy if you need to recharge. (SP6, Police)*

This feeling was not restricted to SP6; a service provider from a women’s shelter (SP12) also expressed similar views, highlighting the importance of setting aside time for dialogue and supervision:

*We’re trying to kind of have a good dialogue with each other, we’re working here together, I think that’s very important. We make sure to set plenty of time to talk to each other and relieve [each other] and colleague tutoring and stuff like that. So, it’s been very good, of course also our recurring supervision, it’s just that being able to group together with our colleagues, with slightly more difficult cases we always try to be two. The two of us have had support contact with a woman who has a lot of needs so we can relieve each other and think together. (SP12, Women’s shelter)*

Similarly, a social worker (SP2) suggested that the presence of competent leadership enhances a sense of confidence in handling IPV cases:

*We have good collegial support, and we have a boss to turn to who is terrifically competent… I always feel like I have someone to call or go to or run a case for or talk to. (SP2, Social work)*

The reliance upon peer networks was perceived as allowing service providers to process emotionally challenging cases. However, SP4 described the emotional toll of their work and how they cope with the perceived inadequately supportive workspace:

*Well, I think it’s the colleagues, and the work partners who are, yes [someone at work I can consult]. And then you develop a little more skin or what I’m going to say is that your skin gets thicker over the years maybe, I don’t know. No, I don’t think I get that [the thick skin, getting used to dealing with the cases]. I go home and shed a few tears, but then you get it... back again. (SP4, Social work)*

Besides colleagues, another source of support was supervisors or managers. Some service providers reported having supportive managers or supervisors, whom they could turn to when colleagues were not available. A healthcare provider (SP5) said:

*If there’s anything, where I don’t have a teammate, I’ll talk to her [my manager]. I feel like I can talk to her. That’s the way it is. If there are colleagues in psychiatry, or the women’s shelter, or someone involved with the same, then you can get help from each other. That’s how it’s been, too*. (SP5, Healthcare)

SP5’s quote not only highlights the support provided by colleagues and managers within the same institution, but also the opportunity to engage in conversations and consultations with colleagues from other institutions – inter-institutional collegial support. This ability to reach out to colleagues from other institutions was perceived as a strength that enhances the support network for service providers.

## Discussion

This study applied an adapted version of Colombini et al.’s [33] conceptual framework on health-system readiness to assess how formal support systems in Sweden respond to women with disabilities subjected to IPV. By analysing indicators of readiness at the structural, organizational, and provider levels, this study has identified barriers and facilitators that shape the capacity of healthcare, social services, shelters, the Centres against Violence, and the police to deliver inclusive and effective IPV support for women with disabilities.

At the structural level, our study reveals that the presence of functional collaboration spaces, both inter-institutional (between different organizations) and intra-institutional (within the same organization), emerged as a key facilitator of readiness. These collaborative spaces were described as facilitating knowledge-sharing and simplifying the coordination of IPV support services. Participants further described regular multidisciplinary meetings and team reflections upon violence against women with disabilities as enabling knowledge-sharing.

However, our results also reveal fragmented and inconsistent collaborative practices. The absence of formalized communication protocols and feedback mechanisms between institutions compromised continuity of support and hindered coordinated responses. This fragmentation is particularly detrimental for women with disabilities, whose needs often span multiple service domains and require integrated, cross-sectoral support.

Our findings on the importance of collaboration in enhancing readiness are corroborated by a study that emphasizes the necessity of collaborations between domestic violence and disability service providers to enhance services and provide care for women with disabilities who experience domestic violence [40]. A qualitative meta-analysis also showed that health practitioners improve their readiness to address domestic violence by collaborating with team members within their organizations and with specialist professionals outside their teams [13]. For instance, practitioners reported feeling more prepared and supported when they could discuss cases and strategies with colleagues and experts [13]. Similarly, in the social-work sector, personal contact and direct communication between social workers and external agencies boosted multi-sectoral collaboration [41]. Furthermore, a systematic review identified interdisciplinary collaboration among formal service providers for women subjected to IPV as essential for providing comprehensive care to IPV victim-survivors [29].

Although these studies did not specifically address IPV response for people with disabilities, they underscore the importance of functional collaborative spaces, which is even more critical when supporting women with disabilities, as our study found. Women with disabilities may have unique needs that require specialized knowledge and coordinated efforts from various support institutions.

Structural barriers that hindered readiness, particularly for professionals in shelters, included unstable funding, workload pressures, and inefficient data systems. These factors had a negative influence on the provision of disability-specific IPV support. Inadequate resources, including time, have been reported in other settings as influencing readiness to respond to domestic violence against women [41].

At the organizational level, our study found notable variation in the availability and specificity of disability-inclusive IPV guidelines. Our findings show that, while both national-level and regional-specific IPV routines do exist [42], their integration of disability-specific needs is inadequate. This inadequacy potentially leads to inconsistencies in providing IPV support services and undermines the ability of service providers to effectively address the intersectional needs of women with disabilities subjected to IPV. Previous research in Sweden supports this finding, showing that the availability of administrative procedures increases social workers’ readiness to inquire about women’s exposure to IPV [10]. Additionally, it has been found that organizational support, including practice protocols and guidelines, is crucial for social workers in responding to victim-survivors of IPV [21].

The current study also found that accessibility barriers constitute a substantial gap in organizational readiness for supporting women with disabilities subjected to IPV, encompassing not only physical inaccessibility but also information and communication barriers. For instance, inaccessible buildings and transport systems prevent women with disabilities from reaching IPV support services. Similarly, the lack of accessible information and communication tools make it difficult for these women to understand and navigate the services available to them, and this in turn influenced the service providers’ readiness to act optimally. A systematic review of barriers on access to healthcare for women with disabilities identified similar issues [30]. Factors such as inaccessible equipment, lack of physical access to transport systems and buildings, lack of access to information and websites not being disability adapted, lack of assistive devices, and insufficient consultation and/or notification were found to impact upon healthcare access for women with disabilities [30].

Furthermore, due to the accessibility challenges, fragmentation of support services was identified as making the navigation of IPV support even more difficult, especially for those with various impairments, including cognitive or intellectual and physical impairments. Fragmentation of services and disjointed referrals between support institutions influence readiness, leading to gaps in support for IPV victim-survivors [19].

At the provider level, our study highlights a widespread recognition among service providers that there is a need for more training specifically focused on supporting women with disabilities subjected to IPV. While general IPV training was relatively common, it was rare for this training to include disability-specific components. As a result, many service providers relied upon personal experience or peer support to manage complex cases (a term used to refer to cases involving women with disabilities, especially those with intellectual disabilities). This reflects a systemic gap in the integration of disability-inclusive practices within IPV support services.

Previous studies have shown that training is closely linked to increased readiness. For instance, Lundberg and Bergmark [10], found that training enhances social workers’ readiness to inquire about IPV [10]. Similarly, Hegarty et al. [13] demonstrated that training on how to identify and respond to IPV among health professionals is crucial [13]. Furthermore, a study on support for persons with disabilities highlighted that Australian disability and mainstream service providers were calling for training and the development of resources to guide professional practice across disability and wider violence-response services [28].

Supportive workplace cultures and collegial networks were identified as vital facilitators of provider readiness. Strategies such as peer consultation, regular supervision, and collaborative case management provided emotional support and practical guidance. These networks also served as spaces for debriefing and processing the emotional toll of IPV work, which is particularly important in cases involving complex and intersectional vulnerabilities.

Leung et al. [16] argue that training interventions should incorporate motivational and emotional components, including strategies for managing vicarious trauma and promoting self-care [16]. In our study, service providers who felt supported by their teams and supervisors described greater confidence in addressing the needs of women with disabilities subjected to IPV.

*Interaction across readiness domains.* Although we present the three readiness domains separately for analytic clarity, our findings indicate that they are interdependent, with limitations at one level propagating to the others. Structural conditions set the outer limits of what organizations and providers could achieve: unstable funding in the shelter sector may have constrained investment in accessible premises and communication tools, appearing at the organizational level as physical inaccessibility and at the provider level as improvisation, with providers accompanying women to appointments and attempting to interpret on their behalf. Similarly, the absence of formalized inter-institutional feedback mechanisms was reflected organizationally in fragmented referral pathways and, for individual providers, as an inability to establish what happened to a woman after referral. Organizational conditions in turn appeared to mediate between structural constraints and provider capacity: where disability-inclusive guidelines were absent, providers fell back on personal experience and peer consultation, so that the low proportion reporting disability-specific training and the widespread reliance on colleagues may be read as two expressions of the same organizational gap. The relationship could also have operated upward, as organizational cultures supporting supervision and multidisciplinary meetings gave providers access to knowledge networks that partially compensated for absent formal training and fed back into the intersectoral collaboration that constitutes structural readiness.

These interactions suggest that readiness indicators should not be read additively, as though a deficit in one domain might be offset by strength in another. Provider competence could not compensate for an inaccessible building or an unavailable interpreter, which suggests that structural and organizational provisions function as preconditions rather than parallel contributors, a proposition warranting explicit testing in future research.

Taken together, our findings suggest that readiness cannot be understood as a matter of individual knowledge and skills alone. It requires the capacity to recognize and respond to the varied needs and experiences of women with disabilities, and this capacity depends as much on the structural and organizational conditions in which providers work as on their own competence. Addressing the barriers women with disabilities face therefore calls for specialized knowledge, accessible and well-resourced services, and a workplace culture that values empathy, collaboration, and continuous learning.

### Implications

Our study identifies several cross‑sector strategies that can strengthen support for women with disabilities subjected to IPV. Firstly, services across the formal support system– including healthcare, social services, the police, and women’s shelters– would benefit from integrating disability‑inclusive IPV inquiry and communication practices into routine procedures. Ensuring that guidelines, interactions, and screening tools are adapted for women with diverse cognitive, sensory, and physical needs is central to improving early identification and support. Secondly, enhancing intersectoral referral pathways through formalized feedback loops and clear protocols could improve the continuity and coordination of care. This is particularly important for women with disabilities, whose support needs often span multiple institutions. Thirdly, organisations across sectors can promote readiness by strengthening accessibility– physical, informational, and communicative – and by providing staff with targeted training that addresses both IPV and disability inclusion. Together, these measures can help healthcare providers and other frontline institutions operationalize the findings of this study and deliver more coordinated, responsive, and disability‑inclusive IPV support.

Although situated within Sweden’s structured and multisectoral welfare system, the findings also carry relevance for international efforts to strengthen disability‑inclusive IPV responses. Sweden’s coordinated service architecture –linking healthcare, social services, shelters, police, and specialised violence centres – demonstrates the value of formalised collaboration and shared accountability in addressing IPV among women with disabilities. Features such as universal access, publicly funded services, and legal frameworks mandating accessibility offer examples of how inclusive practices can be institutionalized. While the organisation of IPV support varies across contexts, the principles highlighted in this study such as integrating disability inclusion into IPV screening/inquiry and referral, ensuring accessibility, and investing in cross‑sector collaborations, can inform global policy efforts to build more responsive, equitable, and coordinated IPV support systems. These insights may be especially valuable for countries seeking to strengthen multisectoral coordination and develop inclusive, survivor‑centred IPV interventions.

### Limitations

Our study has several limitations that should be considered when interpreting the findings.

*Conceptual framework.* We used the readiness framework developed by Colombini et al. [33], which was originally designed to assess health system readiness in low- and middle-income countries. Although the authors suggest that the framework can be adapted for multisectoral use and applied in high-income settings, it was not originally tailored for the Swedish context or for the broader range of institutions included in this study. Despite this, we found the framework useful for assessing indicators of readiness across the formal IPV support institutions: healthcare, social services, shelters, the police, and the Centre against Violence. The framework was however applied primarily as an analytical lens during the data analysis phase, rather than being integrated from the beginning of the study. As a result, our data may not capture all the dimensions of readiness outlined in the framework, and some aspects might have been more visible had the framework guided the development of the interview guides and survey instrument. The findings should therefore be interpreted as addressing those dimensions of readiness that emerged as salient, rather than as a comprehensive account of all factors shaping readiness to support women with disabilities.

*Analytical depth of the quantitative component.* Our survey analysis is descriptive, mapping the distribution of readiness indicators rather than modelling associations between them or testing differences across groups. This reflects both the exploratory design and the nature of the measures: items were developed inductively from the qualitative phase as single, discrete indicators rather than as components of a validated composite scale, and inferential modelling would therefore attribute to them a measurement status they do not possess. While appropriate to the study’s aims, this means we cannot establish which structural or organizational conditions are most strongly associated with provider preparedness, nor quantify the relative contribution of each readiness domain. Developing and validating a multisectoral, disability-inclusive readiness instrument, and using it to examine such associations across sectors and professional groups, is an important direction for future research.

*Sample composition and response rate.* The inclusion of a wide range of support institutions allowed for a broader view of the IPV support system, but it also introduced variation in institutional structures and operational contexts, and the number of participants varied across institutions, which may have influenced the balance of insights from each sector. Nonetheless, we believe that including all relevant institutions was essential for understanding the broader systemic readiness, especially given that previous research has often focused on individual sectors in isolation.

The response rate of 32.9% also warrants closer consideration than non-response bias in the general sense. Participation in a survey concerning IPV and disability is unlikely to be independent of the constructs of interest: providers who are more knowledgeable about IPV, more engaged with disability issues, or working in organizations where these questions are actively discussed may have been more inclined to respond. If so, our figures for training exposure, guideline availability, accessibility provisions, and access to consultation may represent an upper bound rather than a neutral estimate of conditions across the sector, and the low proportions we report, particularly for disability-specific training (18%) and communication tools (19%), may therefore understate the extent of these gaps. We were not able to compare respondents with non-respondents on register characteristics within the scope of this study.

*Nature of the data.* The survey captured providers’ self-reported perceptions of organizational conditions rather than independently verified institutional capacity. Perceived accessibility, perceived availability of guidelines, and perceived preparedness are meaningful indicators in their own right, since providers act on their understanding of what their organization offers. However, they may diverge from documented policy or audited provision in either direction: a provider may be unaware of an existing routine, as illustrated by the shelter participant who did not know that a sign-language interpreter could be requested, or may report a guideline as available without it being consistently applied. Our findings should therefore be understood as describing the readiness environment as providers experience it. Research combining self-report with organizational document review, accessibility audits, and observational assessment would provide a fuller account of institutional readiness. The survey also relied upon participants’ recall of practices over the preceding year, which introduces the potential for recall bias.

*Instrument design, language, and timing.* The design of the survey questionnaire, including the wording of questions and language translations, may have affected how respondents interpreted and answered certain questions, despite undergoing a metrology standard review. Because the interviews were translated from Swedish into English for analysis, some linguistic nuance may not be fully retained, however, the research team worked iteratively between translated material and original Swedish transcripts to ensure that analytic interpretations remained accurate. Finally, the qualitative data were collected during the early phase of the COVID 19 pandemic (April–June 2020), which may have influenced both service delivery and participants’ perceptions of readiness. Although the interviews did not focus explicitly on COVID 19, it is possible that some of the challenges described by participants were amplified by the broader disruptions associated with the pandemic.

## Conclusions

Our study has identified barriers and facilitators across key indicators of readiness to respond to women with disabilities subjected to IPV within Sweden’s formal IPV support systems. At the structural level, while collaborative spaces were found to enhance coordination and knowledge-sharing, fragmented practices and weak feedback mechanisms limited the continuity of support. At the organizational level, the inadequacy of disability-inclusive IPV guidelines and persistent accessibility barriers contributed to inconsistent and often inadequate support. At the provider level, limited access to disability-specific training led many professionals to rely upon personal experience and peer support, highlighting a gap in preparedness. However, supportive workplace cultures and collegial networks emerged as important enablers of provider resilience and readiness.

Aligning with the existing literature, our findings underscore the need for comprehensive targeted training, structured feedback mechanisms, accessibility, and inter-sectoral collaboration to meet the unique and intersecting needs of women with disabilities seeking IPV support. It is essential to address these challenges and build upon existing strengths in order to create a more inclusive and effective support system.

Based on our data, an IPV support system for women with disabilities that was truly ready would be characterized by inclusive policies and guidelines that explicitly address their needs, institutionally supported training programmes with disability-specific content, and well-established mechanisms for collaboration across sectors. It would also ensure physical, informational, and communicative accessibility, while fostering workplace cultures that support emotional well-being and professional development. Ultimately, such a system would adopt an integrated, interdisciplinary approach – bringing together healthcare, social services, shelters, the police, and other relevant institutions – to provide holistic, inclusive, coordinated, and survivor-centred support.
